# A population-based matched cohort study examining the mortality and costs of patients with community-onset *Clostridium difficile* infection identified using emergency department visits and hospital admissions

**DOI:** 10.1371/journal.pone.0172410

**Published:** 2017-03-03

**Authors:** Natasha Nanwa, Beate Sander, Murray Krahn, Nick Daneman, Hong Lu, Peter C. Austin, Anand Govindarajan, Laura C. Rosella, Suzanne M. Cadarette, Jeffrey C. Kwong

**Affiliations:** 1 Leslie Dan Faculty of Pharmacy, University of Toronto, Toronto, Canada; 2 Toronto Health Economics and Technology Assessment Collaborative, Toronto, Canada; 3 Public Health Ontario, Toronto, Canada; 4 Institute of Health Policy, Management and Evaluation, University of Toronto, Toronto, Canada; 5 Institute for Clinical Evaluative Sciences, Toronto, Canada; 6 University Health Network, Toronto, Canada; 7 Department of Medicine, University of Toronto, Toronto, Canada; 8 Sunnybrook Health Sciences Centre, Toronto, Canada; 9 Mount Sinai Hospital, Toronto, Canada; 10 Division of General Surgery, Department of Surgery, University of Toronto, Toronto, Canada; 11 Dalla Lana School of Public Health, University of Toronto, Toronto, Canada; 12 Department of Family and Community Medicine, University of Toronto, Toronto, Canada; Cleveland Clinic, UNITED STATES

## Abstract

Few studies have evaluated the mortality or quantified the economic burden of community-onset *Clostridium difficile* infection (CDI). We estimated the attributable mortality and costs of community-onset CDI. We conducted a population-based matched cohort study. We identified incident subjects with community-onset CDI using health administrative data (emergency department visits and hospital admissions) in Ontario, Canada between January 1, 2003 and December 31, 2010. We propensity-score matched each infected subject to one uninfected subject and followed subjects in the cohort until December 31, 2011. We evaluated all-cause mortality and costs (unadjusted and adjusted for survival) from the healthcare payer perspective (2014 Canadian dollars). During our study period, we identified 7,950 infected subjects. The mean age was 63.5 years (standard deviation = 22.0), 62.7% were female, and 45.0% were very high users of the healthcare system. The relative risk for 30-day, 180-day, and 1-year mortality were 7.32 (95% confidence interval [CI], 5.94–9.02), 3.55 (95%CI, 3.17–3.97), and 2.59 (95%CI, 2.37–2.83), respectively. Mean attributable cumulative 30-day, 180-day, and 1-year costs (unadjusted for survival) were $7,434 (95%CI, $7,122-$7,762), $12,517 (95%CI, $11,687-$13,366), and $13,217 (95%CI, $12,062-$14,388). Mean attributable cumulative 1-, 2-, and 3-year costs (adjusted for survival) were $10,700 (95%CI, $9,811-$11,645), $13,312 (95%CI, $12,024-$14,682), and $15,812 (95%CI, $14,159-$17,571). Infected subjects had considerably higher risk of all-cause mortality and costs compared with uninfected subjects. This study provides insight on an understudied patient group. Our study findings will facilitate assessment of interventions to prevent community-onset CDI.

## Introduction

*Clostridium difficile* infection (CDI) has been deemed “a leading cause of patient harm” by the Centers for Disease Control and Prevention with 453,000 infections, 83,000 recurrences, and 29,300 deaths estimated in the United States in 2011 [1, 2]. Approximately 29% to 58% of CDI cases have symptom onset in the community or within two or three days of a hospital admission [3–6]. These cases are classified as having community-onset CDI, regardless of whether infection originated from a healthcare facility or the community [4]. Few studies have evaluated clinical or cost outcomes in subjects with community-onset CDI [7], therefore the objective of this study was to determine the risk of all-cause mortality and healthcare costs associated with community-onset CDI.

## Materials and methods

### Study design, setting and subjects

We conducted a population-based propensity-score matched cohort study of individuals with community-onset CDI to examine attributable mortality and costs. The latter was determined using an incidence-based cost-of-illness approach [8] from the perspective of the Ontario Ministry of Health and Long-Term Care (healthcare payer). This approach measures long-term costs of newly diagnosed cases from an index date (e.g., diagnosis) to a defined point in time (e.g., post one year, death) in contrast to the prevalence-based approach, which evaluates the cost of disease (across varying disease stages) during a defined time period (e.g., 1 year) [8–11]. We conducted this study at the Institute for Clinical Evaluative Sciences (ICES) in Toronto, Canada, which houses provincial health administrative data on publicly funded healthcare that are linkable at the individual level using encoded unique identifiers [12]. This study was approved by the Sunnybrook Health Sciences Centre Institutional Review Board and the University of Toronto Office of Research Ethics. Written or verbal informed consent was not required from the subjects in this study, as ICES is permitted to obtain and use personal health information without patient consent for specific purposes, as outlined in Ontario’s Personal Health Information Protection Act.

We identified incident cases of community-onset CDI (symptomatic infected subjects) using the International Statistical Classification of Diseases and Related Health Problems, 10th Revision, Canada (ICD-10-CA) code A04.7 (enterocolitis due to CDI). To be classified as community-onset, this code had to be present (between January 1, 2003 and December 31, 2010) during the following healthcare encounters: a) an emergency department (ED) visit (index date: ED visit date or physician office date if CDI-related symptoms documented within two weeks prior); b) a non-elective hospital admission with CDI as the principal diagnosis and a colectomy (see S1 Table for the intervention codes for identifying colectomies) within two days of hospitalization (index date: admission date); c) a non-elective hospital admission lasting ≤2 days (index date: admission date); or d) a non-elective hospital admission with a principal diagnosis of CDI with CDI-related symptoms documented within two weeks prior to admission (index date: physician office or ED visit date). CDI-related symptoms were defined as a physician office visit coded as abdominal pain or diarrhea; or an ED visit coded as cramps, abdominal pain, diarrhea, or suspicious CDI (see S2 Table for codes used to identify these conditions). For infected subjects that had more than one healthcare encounter (i.e., a to d), the earliest encounter was used. We were unable to identify infected subjects using physician office visits alone, as there is no code specific to CDI amongst the diagnostic codes used for Ontario physician billing claims. As a result, our study population likely includes predominantly subjects with more severe infection. The entire cohort was followed until December 31, 2011.

We used propensity-score matching without replacement to match each infected subject to one randomly selected (hospitalized or community dwelling) uninfected subject (those without a record for ICD-10-CA code A04.7). The index dates for the uninfected subjects were randomly assigned between January 1, 2003 and December 31, 2010. Using a logistic regression model that regressed infection status (infected versus uninfected), the propensity score was calculated based on covariates at the index date (neighborhood income quintile, rurality), covariates within the previous 12 weeks before the index date (healthcare utilization, a record of an infection that could have led to a prescription for an antibiotic), and covariates within the two years prior to the index date (comorbidities using the John Hopkins Adjusted Clinical Groups System^®^ which measures an individual’s health resource utilization by assigning their ICD codes into diagnosis groups, e.g., Aggregated Diagnosis Groups, Resource Utilization Bands [13–15]). See S3 Table for additional details on these covariates.

To evaluate the effect of CDI on costs prior to death, we re-matched without replacement each infected subject who died during the observation period (between 2003 and 2011) to one randomly selected uninfected subject who also died during the observation period. Using a logistic regression model that regressed infection status (infected versus uninfected), the propensity score was calculated based on neighborhood income quintile, rurality, and co-morbidities, measured three months before death.

For both matching approaches, subjects were matched on the logit of the propensity score using calipers of width equal to 0.2 of the standard deviation of the logit of the propensity score [16]. Subjects were also hard matched on sex, birth year ±3 years, and date (index date for the initial match and three months prior to death for the re-match) ±30 days. The balance of measured baseline covariates were assessed using standardized differences, with standardized differences <0.1 indicating negligible differences between the matched infected and uninfected subjects in terms of the covariates included in the propensity score matches [17].

### Outcomes

We examined 30-day, 180-day, and 1-year all-cause mortality. We evaluated the costs of publicly funded healthcare services (e.g., inpatient hospitalization, ED visits, physician services) [18], expressed as total costs unadjusted for survival (30-day, 180-day, and 1-year costs) and adjusted for survival (1-, 2-, and 3-year costs).

### Analysis

Using the matched sample, we estimated the difference in the probability of death (assessing its statistical significance using McNemar’s test) and the number needed to harm (NNH, reciprocal of the absolute difference in the probability of death), along with the relative risk (RR) and 95% confidence intervals (CI) for all-cause mortality [19, 20]. We also graphed the 3-year survival for the matched infected and uninfected subjects.

We calculated all cost outcomes using the matched sample, and reported costs in 2014 Canadian dollars ($1 Canadian = $0.9054 US [21]). Attributable cumulative costs unadjusted for survival were evaluated by determining the mean difference between the matched infected and uninfected subjects. To determine 95%CIs we employed bootstrapping methods [22]. Lastly, we stratified costs by sex, age group, diagnosis year, and those who died within one year of their index date.

We determined attributable cumulative costs adjusted for survival using the phase-of-care approach. This method involves organizing an individual’s observation time into phases that represent patient care from diagnosis to death and is consistent with *C*. *difficile* disease history [23–25]. We defined three phases of care: acute infection (lasting up to 6 months), continuing care (up to 110 months), and final care (lasting up to 3 months). We allocated each subject’s observation time to each phase of care in the following order: final care, acute infection, and continuing care. For example, for subjects with 12 months of observation time (index date to death date), the last 3 months were allocated to final care, the first 6 months were allocated to acute infection, and the intervening period (3 months) was allocated to continuing care. For subjects with 2 months of observation time (index date to death date), the entire time was allocated to final care.

Phase-specific costs were measured in 30-day intervals [24]. We calculated attributable phase-specific costs by evaluating the mean difference between the matched infected and uninfected subjects. We used bootstrapping methods to determine the 95%CIs [22]. Further, phase-specific costs were stratified by healthcare services (e.g., physician services, hospitalizations) to understand resource use over the course of the disease. To determine attributable cumulative mean 1-, 2-, and 3-year costs (and 95% CIs), the mean attributable phase-specific costs (acute infection, continuing care, and final care) were combined with the survival data as described by Yabroff et al. [24]. Costs calculated beyond one year were discounted at 5% annually [26]. Lastly, we stratified the 1-, 2-, and 3-year costs by sex, age group, and those who died within one year of their index date.

### Sub-analysis

As a sub-analysis, among the matched cohort, we stratified the cost outcomes of those who had an ED visit as their first healthcare encounter, into those who were discharged home versus admitted to hospital.

## Results

### Study cohort

We identified 7,950 subjects with community-onset CDI between January 1, 2003 and December 31, 2010 (22% of all individuals with a CDI code in Ontario between 2003 and 2010, n = 36,258). Half (50.2%) had a CDI-coded ED visit (with 1,728 being admitted to hospital), 0.6% had a non-elective CDI-coded hospitalization with a colectomy conducted within two days of admission, 13.8% had a non-elective CDI-coded hospitalization lasting ≤2 days, and 35.4% had a non-elective CDI-coded hospitalization and CDI-related symptoms within two weeks prior to admission.

The estimated crude annual mean incidence of community-onset CDI was 7.8 per 100,000 population. The mean age was 63.5 years (standard deviation = 22.0), 62.7% were female, and 45.0% were very high users of the healthcare system at index date. Nearly three-quarters (72.4%) had healthcare utilization and nearly half (47.5%) had an infection prompting antibiotic therapy within the previous 12 weeks before the index date (Table 1). All subjects had between 1 and 9 years of follow-up if they did not die.

**Table 1:** Selected characteristics prior to matching.

	Community-onset CDI (n = 7,950)
**Age**^a^**, mean±SD, median**	63.5±22.0, 69 years
**Female, %**	62.7
**Age group**^a^**, %**	
**Children (≤18 years)**	4.1
**Adults (19–64 years)**	38.6
**Older adults (≥65years)**	57.3
**Crude annual incidence rate**^b^**, per 100,000 population**	
**2003**	4.9
**2004**	6.7
**2005**	8.2
**2006**	7.4
**2007**	8.9
**2008**	9.4
**2009**	8.7
**2010**	8.4
**Neighborhood income quintile**^a^**, %**	
**1 (lowest)**	22.0
**2**	20.5
**3**	19.0
**4**	19.2
**5 (highest)**	19.3
**Rurality**^a^**, %**	
**Major urban**	59.9
**Non-major urban**	29.0
**Rural**	11.1
**Very high users of the healthcare system**^c^**, %**	45.0
**Healthcare utilization within 12 weeks prior to index date, %**	72.4
**Possible prescription for an antibiotic within 12 weeks prior to index date, %**	47.5
**All-cause mortality**^d^**, %**	
**30 day**	11.8
**180 day**	19.6
**1 year**	23.0
**End of study period**	35.8
**Costs unadjusted for survival**^d^**, mean, median**	
**30 day**	$9,535, $6,324
**180 day**	$21,488, $9,754
**1 year**	$28,782, $12,705

^a^Measured at the index date.

^b^Not standardized to a specific year.

^c^Measure of co-morbidities (within the two years prior to the index date, see S3 Table).

^d^Post index date.

CDI—*C*.*difficile* infection. SD—standard deviation.

### Matching results

We matched 81.0% (n = 6,437) of the infected subjects to uninfected subjects (Table 2). Thirty-four percent (n = 2,158) of the matched infected subjects died during the study period (January 2003 to December 2011). In the re-match, we matched 99.2% (n = 2,140) of these cases to similar uninfected subjects who also died (S4 Table). For both sets of matches, all standardized differences were <0.1.

**Table 2:** Selected characteristics of those with community-onset CDI before and after matching.

	Infected subjects	Pool of uninfected subjects	Standardized differences comparing infected subjects and pool of uninfected subjects	Unmatched infected subjects	Matched infected subjects	Matched uninfected subjects	Standardized differences comparing matched infected and uninfected subjects
**n**	7,950	512,184	NA	1,513	6,437	6,437	NA
**Hard match variables**^a^							
**Age, mean±SD**	63.5±22.0	57.7±21.0	0.27	62.4±24.1	63.8±21.4	64.2±21.0	0.02
**Female, %**	62.7	62.2	0.01	52.2	65.1%	65.1%	0.00
**First healthcare encounter, %**							
**CDI-coded ED visit**	50.2	NA	NA	47.9	50.7	NA	NA
**CDI-coded non-elective hospital admission**	49.8	NA	NA	52.1	49.3	NA	NA
**Propensity score variables**							
**Neighborhood income quintile**^a^**, %**							
**1 (lowest)**	22.0	20.0	0.05	22.5	21.9	20.7	0.03
**2**	20.5	20.4	0.00	21.1	20.4	22.2	0.04
**3**	19.0	19.5	0.01	18.8	19.1	19.1	0.00
**4**	19.2	19.7	0.01	18.5	19.3	19.2	0.00
**5 (highest)**	19.3	20.3	0.03	19.2	19.3	18.9	0.01
**Rurality**^a^**, %**							
**Major urban**	59.9	70.4	0.22	56.2	60.8	60.1	0.01
**Non-major urban**	29.0	21.9	0.16	31.7	28.4	28.2	0.00
**Rural**	11.1	7.7	0.12	12.1	10.8	11.7	0.03
**Very high users of the healthcare system**^b^**, %**	45.0	8.8	0.90	69.7	39.2	38.4	0.02
**Aggregated Diagnosis Groups- specific to infection**^b^**, %**							
**Time Limited: Minor- primary infections**	79.2	42.3	0.82	92.1	76.2	73.6	0.06
**Time Limited: Major- primary infections**	35.8	9.0	0.68	66.8	28.5	28.8	0.01
**Likely to Recur: Discrete- infections**	42.8	19.0	0.53	52.5	40.5	39.6	0.02
**Healthcare utilization**^c^**, %**	72.4	11.6	1.56	99.6	66.0	65.1	0.02
**Record of an infection that may have led to an antibiotic prescription**^c^**, %**	47.5	9.6	0.92	82.2	39.4	36.0	0.07

^a^Measured at the index date.

^b^Measure of co-morbidities (within the two years prior to the index date, see S3 Table).

^c^Measured within the previous 12 weeks before the index date.

CDI–*C*.*difficile* infection. NA—not applicable. SD—standard deviation.

### Outcomes

**Fig 1:**
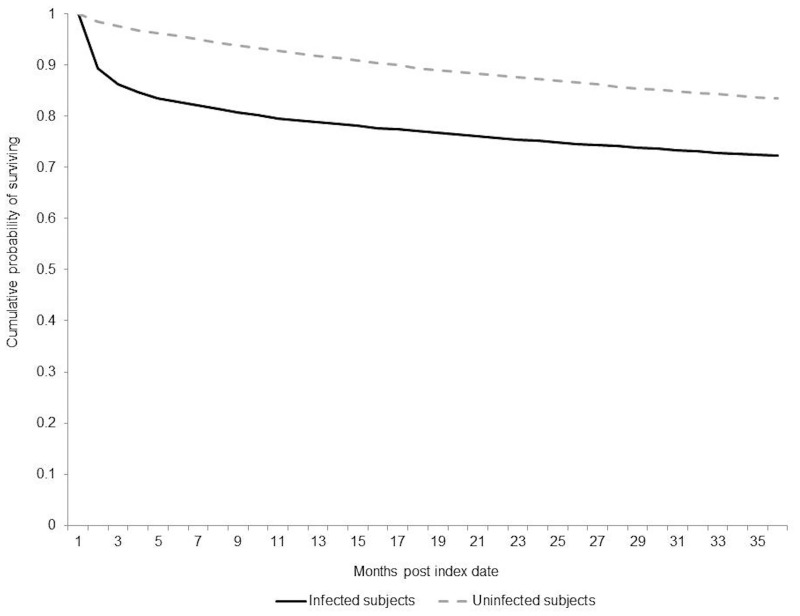
Survival curve for infected subjects and their matched pairs, three years post index date.

Community-onset CDI was associated with increased risk of all-cause mortality among infected subjects compared to uninfected subjects (Table 3). The absolute differences in the risk of 30-day, 180-day, and 1-year mortality were 9.2%, 12.9%, and 13.0%, respectively (p<0.0001), for a NNH for 1-year mortality of 8. The RRs for 30-day, 180-day and 1-year mortality were 7.32 (95%CI, 5.94–9.02), 3.55 (95%CI, 3.17–3.97), and 2.59 (95%CI, 2.37–2.83), respectively. Lower survival was also observed among infected subjects three years after the index date (Fig 1).

**Table 3:** All-cause mortality and healthcare costs attributable to community-onset CDI.

	n matched pairs	Infected subjects	Uninfected subjects	Attributable outcome (95% CI)
**All-cause mortality**^a^				
**30 days**	6,437	10.7%	1.5%	9.2%, RR = 7.32 (5.94–9.02)
**180 days**	6,437	17.9%	5.0%	12.9%, RR = 3.55 (3.17–3.97)
**1 year**	6,437	21.2%	8.2%	13.0%, RR = 2.59 (2.37–2.83)
**Mean cost outcomes unadjusted for survival**^a^				
**30-day cumulative costs**	6,437	$9,234, Median = $6,130	$1,800, Median = $240	$7,434 ($7,122-$7,762), Median = $5,891
**180-day cumulative costs**	6,437	$20,376, Median = $9,154	$7,859, Median = $1,760	$12,517, ($11,687-$13,366), Median = $7,394
**1-year cumulative costs**	6,437	$27,209, Median = $11,900	$13,992, Median = $3,817	$13,217 ($12,062-$14,388), Median = $8,083
**Mean cost outcomes by phase**^b^				
**Acute infection costs**	5,454	$3,503	$1,209	$2,294 ($2,135-$2,463)
**Continuing care costs**	5,160	$979	$478	$502 ($435-$573)
**Final care costs**^c^	2,140	$13,658	$10,560	$3,099 ($2,364-$3,823)
**Mean cost outcomes adjusted for survival**^a^				
**1-year cumulative costs**	6,437	NA	NA	$10,700 ($9,811-$11,645)
**2-year cumulative costs**				
**Undiscounted**	6,437	NA	NA	$13,312 ($12,024-$14,682)
**Discounted 5%**	6,437	NA	NA	$12,646 ($11,423-$13,948)
**3-year cumulative costs**				
**Undiscounted**	6,437	NA	NA	$15,812 ($14,159-$17,571)
**Discounted 5%**	6,437	NA	NA	$15,022 ($13,451-$16,692)

^a^Post index date.

^b^Costs standardized to 30 days.

^c^Costs derived from the re-matched infected subjects who died (S4 Table).

CI—confidence interval. NA—not applicable. RR—relative risk.

Community-onset CDI was associated with 1.9- to 5.1-fold higher mean costs compared to uninfected subjects, up to one year post index date (Table 3). Mean attributable cumulative 30-day, 180-day, and 1-year costs per subject unadjusted for survival were $7,434 (95%CI, $7,122-$7,762), $12,517 (95%CI, $11,687-$13,366), and $13,217 (95%CI, $12,062-$14,388), respectively. Mean attributable cumulative 1-, 2-, and 3-year costs per subject adjusted for survival (undiscounted) were $10,700 (95%CI, $9,811-$11,645), $13,312 (95%CI, $12,024-$14,682), and $15,812 (95%CI, $14,159-$17,571), respectively.

**Fig 2:**
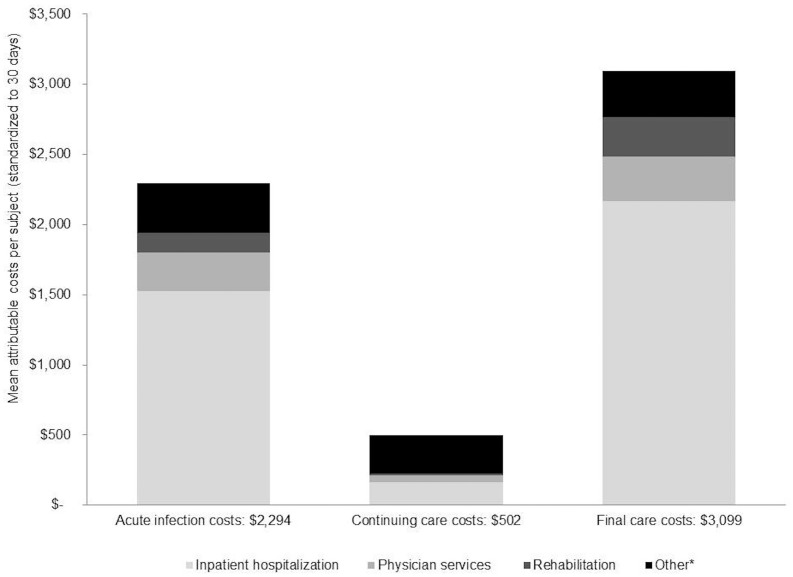
Stratification of phase-specific costs by healthcare services. *Other- Includes same day surgery, ED visits, outpatient medication, non-physician services, outpatient laboratory tests, complex continuing care, home care services, long-term care, dialysis clinic visits, cancer clinic visits, and assisted devices.

In the stratified analyses, hospitalizations and physician visits comprised the largest cost components across all phases (Fig 2). Mean attributable costs were higher among those ≥65 years of age, those infected in 2008, and those who died within one year post index date (S5 Table).

### Sub-analysis

Among the matched cohort (n = 6,437), 50.7% (n = 3,266) had an ED visit as their first healthcare encounter (Table 2). From this group, 58.8% (n = 1,920) were discharged home and 41.2% (n = 1,346) were admitted to hospital. Mean attributable costs were higher among those admitted to hospital versus those discharged home (Table 4).

**Table 4:** Stratified mean attributable costs per subject among those that had their first healthcare encounter as an ED visit.

	n matched pairs	Costs unadjusted for survival			Costs adjusted for survival		
		Cumulative 30-day costs (95% CI)	Cumulative180-day costs (95% CI)	Cumulative1-year costs (95% CI)	Cumulative 1-year costs, undiscounted (95% CI)	Cumulative 2-year costs, undiscounted (95% CI)	Cumulative 3-year costs, undiscounted (95% CI)
**Overall**	3,266	$5,280 ($4,842-$5,733)	$9,458 ($8,387-$10,560)	$10,229 ($8,801-$11,724)	$8,517 ($7,293- $9,826)	$11,156 ($9,343- $13,071)	$13,619 ($11,267- $16,090)
**Discharged home**	1,920	$967 ($676-$1,239)	$2,406 ($1,495-$3,334)	$2,610 ($1,141-$4,042)	$2,839 ($1,649- $4,100)	$4,578 ($2,647- $6,718)	$6,229 ($3,595- $9,200)
**Admitted to hospital**	1,346	$11,433 ($10,544-$12,384)	$19,517 ($17,281-$21,872)	$21,096 ($18,013-$24,268)	$13,777 ($11,669- $16,059)	$16,842 ($13,948- $19,988)	$19,551 ($15,983- $23,438)

## Discussion

Infected subjects had 2.6- to 7.3-fold greater all-cause mortality and 1.9- to 5.1-fold greater costs compared to uninfected subjects up to one year post index date. Attributable mortality and healthcare costs were greatest close to the index date. However, differences in mortality and costs still existed three years post index date. This accumulation of costs could be due to CDI recurrences, treatment failure, need for a colectomy, or CDI increasing susceptibility for other conditions. Hospitalization was the largest cost component during the course of the disease, and mean attributable costs were higher among those aged ≥65 years, those infected in 2008 (which could be due to a virulent CDI strain in Ontario that year [27]), and those who died within one year post index date.

Our results were generally similar to published literature, where it has been reported that those with community-onset CDI have a median age of 61 years, 52% to 61% are female, and 11% die within 30 days [3, 28]. In our cohort, the median age was 69 years, 63% were female, and 12% died within 30 days. No previous studies have evaluated attributable mortality among those with community-onset CDI. However, Karas et al. reported a pooled attributable mortality for CDI within 3 months post diagnosis (mostly in hospital settings) of 6.0% overall (8.0% if only including studies published after 2000), whereas we report an attributable mortality of 9.2% at 30 days, 12.9% at 180 days, and 13.0% at 1 year [29]. One explanation for this difference could be a circulating CDI strain that caused more severe disease in Ontario during our study period. Compared to matched cohort studies focused on other conditions, attributable mortality for community-onset CDI in our cohort was comparable or lower [30, 31]. For example, Lash et al. found 180-day mortality for patients hospitalized for chronic obstructive pulmonary disease to be 16.0%, compared to 2.4% in matched uninfected patients (difference = 13.6%), whereas Su et al. found 1-year mortality for those with healthcare-associated *Staphylococcus aureus* infection to be 59.5%, compared to 39.3% in matched uninfected patients (difference = 20.2%) [30, 31].

In terms of economic burden, we could not compare our results to published literature. Sammons et al. did not examine attributable costs beyond a hospital stay for those with community-onset CDI [32]. Kuntz et al. did not present non-attributable costs exclusively for community-onset CDI; instead they presented costs for those identified with CDI in outpatient versus inpatient settings [33]. However, in contrast to Nanwa et al. who conducted a systematic review of the CDI costing literature, the mean attributable 30-day costs among our cohort ($7,434) were lower than their reported range of mean attributable CDI costs for hospitalized patients over a hospital stay ($10,961 to $36,960 [converted to Canadian dollars [34]]) [7]. This difference is likely because not all subjects in our cohort were hospitalized.

Our study had some limitations. We were not able to identify subjects who only had physician office visits for CDI, likely overestimating mortality among those with community-onset CDI and the economic burden of community-onset CDI per subject, as we likely captured more severe cases in our study. However, on a population level we are likely underestimating the total economic impact of community-onset CDI as not all cases are captured. We are uncertain about how many infected subjects we missed; however, Hirshon et al. found that 4% of outpatients with diarrhea tested positive for CDI between 2002 and 2007 in the US [35]. Missing subjects who only visited physician offices for CDI could explain why we captured fewer individuals with community-onset CDI than previously reported (22% versus 29% to 58% of all CDI cases [3, 4]). However, this could also be due to regional variations. Our sub-analysis of matched infected subjects who had an ED visit as their first encounter, provides some insight on the mean attributable costs of mild versus severe community-onset CDI (Table 4).

Additional uncertainly surrounds the validity of our case definition. A Canadian study found a high sensitivity (88%) and specificity (100%) associated with the ICD-9/ICD-10-CA code for CDI when compared to CDI stool toxin results in a cohort of hospitalized ulcerative colitis patients [36]. However, 50.2% of the infected subjects in our study were identified using ED visits and to the best of our knowledge, there are no published CDI validation studies among ED patients. Another study limitation was that our index dates might not represent the date of symptom onset, as this is difficult to establish with a retrospective study design and without CDI-specific laboratory data. Due to this uncertainty we did not stratify our results by where the infection may have originated (healthcare facility or community setting). However, we provide the overall costs of community-onset CDI, which is understudied in the literature and it can be assumed that the majority of subjects captured in our study were healthcare facility-associated cases, since 66.0% of the matched infected subjects had healthcare utilization within the previous 12 weeks before the index date (Table 2). Lastly, 19% of the cohort could not be matched. This proportion of infected subjects represented males and those with greater co-morbidities. Not including the latter could have led to an underestimation of mortality and costs.

The main strengths of our study were the population-based sample, allowing for our results to be generalizable and our access to individually linked datasets, which allowed us to: a) create a comprehensive algorithm to identify infected subjects with community-onset CDI; b) use and define numerous covariates in our matches; and c) incorporate a broad range of healthcare services that are publicly funded in our cost analyses.

Future studies should contrast the burden of healthcare facility-onset CDI to community-onset CDI and attempt to stratify results by where the infection may have been acquired. Also, patient out-of-pocket costs and productivity losses of those with CDI would be useful to further our understanding of CDI burden.

## Conclusions

This study demonstrates that community-onset CDI is associated with increased risk of all-cause mortality and both short- and long-term economic burden. Our estimates of absolute and relative mortality and costs highlight the need for interventions to prevent community-onset CDI.

## Supporting information

S1 Table Canadian Classification of Health Interventions (CCI) codes related to a colectomy procedure. (DOCX)

S2 Table ICD-10-CA and ontario health insurance plan diagnosis codes related to common *C*. *difficile* symptoms. ICD-10-CA—International Statistical Classification of Diseases and Related Health Problems, 10th Revision, Canada. OHIP—Ontario Health Insurance Plan.
(DOCX)

S3 Table Covariates. ^a^For the re-match, the index date was 3 months prior to death. ^b^12 weeks was chosen since studies have found that the onset of CDI symptoms can occur up to 3 months after discharge from a hospital or stopping an antibiotic [6–8] (refer to the references listed with S3 Table). ICD-10- CA—International Statistical Classification of Diseases and Related Health Problems, 10th Revision, Canada. OHIP—Ontario Health Insurance Plan.
(DOCX)

S4 Table Selected details on the re-matched infected subjects who died. ^a^Measured at the index date. ^b^Highest resource utilization band measured within the previous two years before the index date (Adjusted Clinical Groups^®^ were used to measure comorbidities- see S3 Table). CDI–*C*.*difficile* infection. NA—not applicable. SD—standard deviation.
(DOCX)

S5 Table Overall and stratified mean attributable costs per subject. Confidence intervals not reported, however, none of the confidence intervals crossed zero. CDI—C.*difficile* infection. NA—not applicable.
(DOCX)
