# Supplementary material for: A population-based matched cohort study examining the mortality and costs of patients with community-onset Clostridium difficile infection identified using emergency department visits and hospital admissions
Source: PLoS One. 2017 Mar 3;12(3):e0172410. doi: 10.1371/journal.pone.0172410 (PMC5336215; doi:10.1371/journal.pone.0172410)
Supplement: S1 Table — (DOCX) [file pone.0172410.s001.docx]

| **Intervention** | **CCI code** |
| --- | --- |
| Excision partial, large intestine | 1NM87^^ (except 1NM87BA) |
| Excision total, large intestine | 1NM89^^ |
| Excision radical, large intestine | 1NM91^^ |
| Excision partial, rectum | 1NQ87^^ (except 1NQ87BA) |
| Excision total, rectum | 1NQ89^^ |
| Excision total with reconstruction, rectum | 1NQ90^^ |
