# Supplementary material for: A population-based matched cohort study examining the mortality and costs of patients with community-onset Clostridium difficile infection identified using emergency department visits and hospital admissions
Source: PLoS One. 2017 Mar 3;12(3):e0172410. doi: 10.1371/journal.pone.0172410 (PMC5336215; doi:10.1371/journal.pone.0172410)
Supplement: S2 Table — ICD-10-CA—International Statistical Classification of Diseases and Related Health Problems, 10th Revision, Canada. OHIP—Ontario Health Insurance Plan. (DOCX) [file pone.0172410.s002.docx]

| **ICD-10-CA code** | **OHIP code** | **Description** |
| --- | --- | --- |
| C. *difficile* infection |  |  |
| A047 |  | Enterocolitis due to *Clostridium difficile* (if problem prefix flagged as suspicious/questionable) |
| Diarrhea |  |  |
|  | 009 | Diarrhea, gastro-enteritis, viral gastro-enteritis |
| A09 |  | Diarrhea and gastroenteritis of presumed infectious origin |
| A090 |  | Other and unspecified gastroenteritis and colitis of infectious origin |
| A099 |  | Gastroenteritis and colitis of unspecified origin |
| P782 |  | Noninfective neonatal diarrhea |
| K580 |  | Irritable bowel syndrome with diarrhea |
| K591 |  | Functional diarrhoea |
| K529 |  | Noninfective gastroenteritis and colitis, unspecified |
| Abdominal pain |  |  |
|  | 787 | Anorexia, nausea and vomiting, heartburn, dysphagia, hiccough, hematemesis, jaundice, ascites, abdominal pain, melena, masses |
| R100 |  | Acute abdomen |
| R1010 |  | Right upper quadrant pain |
| R1011 |  | Left upper quadrant pain |
| R1012 |  | Epigastric pain |
| R1019 |  | Upper abdominal pain, unspecified |
| R102 |  | Pelvic and perineal pain |
| R1030 |  | Right lower quadrant pain |
| R1031 |  | Left lower quadrant pain |
| R1032 |  | Periumbilical pain |
| R1039 |  | Lower abdominal pain, unspecified |
| R104 |  | Other and unspecified abdominal pain |
| Cramps |  |  |
| R252 |  | Cramp and spasm |
