# Supplementary material for: A population-based matched cohort study examining the mortality and costs of patients with community-onset Clostridium difficile infection identified using emergency department visits and hospital admissions
Source: PLoS One. 2017 Mar 3;12(3):e0172410. doi: 10.1371/journal.pone.0172410 (PMC5336215; doi:10.1371/journal.pone.0172410)
Supplement: S3 Table — aFor the re-match, the index date was 3 months prior to death. b12 weeks was chosen since studies have found that the onset of CDI symptoms can occur up to 3 months after discharge from a hospital or stopping an antibiotic [6–8] (refer to the references listed with S3 Table). ICD-10- CA—International Statistical Classification of Diseases and Related Health Problems, 10th Revision, Canada. OHIP—Ontario Health Insurance Plan. (DOCX) [file pone.0172410.s003.docx]

| **Covariates** | **Details** |
| --- | --- |
| Neighborhood income quintile | - Based on 2006 Canadian Census data [[1](#_ENREF_1)] - Measured at the index date^a^ |
| Rurality | - Obtained from the Rurality Index of Ontario from 2004 and 2008 [[2](#_ENREF_2), [3](#_ENREF_3)] - Measured at the index date^a^ |
| Co-morbidities | - Utilized the John Hopkins Adjusted Clinical Groups® System Aggregated Diagnosis Groups [[4](#_ENREF_4), [5](#_ENREF_5)] - Measured within the 2 years of the index date^a^ |
| Healthcare utilization | - Defined as the presence of any of the following within the 12 weeks prior to the index date^a,b^:   - Emergency department visit   - Hospital admission   - Same-day surgery   - Long-term care stay |
| Possible prescription for an antibiotic | - Determined by the presence of a condition (determined by ICD-10-CA and/or OHIP code) within the 12 weeks prior to the index date^a,b^ that would commonly be prescribed an antibiotic - List of conditions available upon request |

**References for S3 Table**

1. Statistics Canada. Income and Earnings Reference Guide, 2006 Census. 2011 [August 21 2014]; Available from: <http://www12.statcan.ca/census-recensement/2006/ref/rp-guides/income-revenu-eng.cfm>.
2. Kralj B. Measuring ‘rurality’ for purposes of health-care planning: an empirical measure for Ontario. : Ontario Medical Association Economics Department 2000.
3. Kralj B. Measuring Rurality - RIO2008_BASIC: Methodology and Results. Ontario Medical Association Economics Department; 2009 [August 21 2014]; Available from: <https://www.oma.org/Resources/Documents/2008RIO-FullTechnicalPaper.pdf>.
4. The Johns Hopkins University. The Johns Hopkins ACG® System. 2013 [August 21 2014]; Available from: <http://acg.jhsph.org>.
5. University of Manitoba. Concept: Adjusted Clinical Groups® (ACG®) - Overview. 2014 [August 21 2014]; Available from: <http://mchp-appserv.cpe.umanitoba.ca/viewConcept.php?conceptID=1304>.
6. Dubberke ER, McMullen KM, Mayfield JL, Reske KA, Georgantopoulos P, Warren DK, et al. Hospital-associated Clostridium difficile infection: is it necessary to track community-onset disease? Infect Control Hosp Epidemiol. 2009 Apr;30(4):332-7.
7. Dubberke ER, Reske KA, Yan Y, Olsen MA, McDonald LC, Fraser VJ. Clostridium difficile--associated disease in a setting of endemicity: identification of novel risk factors. Clin Infect Dis. 2007 Dec 15;45(12):1543-9.
8. Hensgens MP, Goorhuis A, Dekkers OM, Kuijper EJ. Time interval of increased risk for Clostridium difficile infection after exposure to antibiotics. J Antimicrob Chemother. 2012 Mar;67(3):742-8.
