# Supplementary material for: A population-based matched cohort study examining the mortality and costs of patients with community-onset Clostridium difficile infection identified using emergency department visits and hospital admissions
Source: PLoS One. 2017 Mar 3;12(3):e0172410. doi: 10.1371/journal.pone.0172410 (PMC5336215; doi:10.1371/journal.pone.0172410)
Supplement: S4 Table — aMeasured at the index date. bHighest resource utilization band measured within the previous two years before the index date (Adjusted Clinical Groups® were used to measure comorbidities- see S3 Table). CDI–C.difficile infection. NA—not applicable. SD—standard deviation. (DOCX) [file pone.0172410.s004.docx]

|  | **Infected subjects** | **Pool of uninfected subjects** | **Standardized differences** | **Matched infected subjects** | **Matched uninfected subjects** | **Standardized differences** |
| --- | --- | --- | --- | --- | --- | --- |
| **n** | 2,146 | 817,620 | NA | 2,140 | 2,140 | NA |
| **Hard match variables^a^** |  |  |  |  |  |  |
| **Age, mean±SD** | 79.5±11.0 | 74.8±17.1 | 0.33 | 79.6±10.8 | 79.6±10.8 | 0.00 |
| **Female, %** | 60.1 | 50.2 | 0.20 | 60.1 | 60.1 | 0.00 |
| **Propensity score variables** |  |  |  |  |  |  |
| **Neighborhood income quintile^a^, %** | 23.9 | 23.5 | 0.01 | 23.9 | 24.3 | 0.01 |
| **1 (lowest)** | 21.1 | 21.3 | 0.01 | 21.1 | 21.5 | 0.01 |
| **2** | 20.1 | 19.1 | 0.03 | 20.1 | 19.1 | 0.03 |
| **3** | 17.6 | 18.5 | 0.02 | 17.6 | 17.2 | 0.01 |
| **4** | 17.3 | 17.7 | 0.01 | 17.3 | 17.8 | 0.01 |
| **5 (highest)** |  |  |  |  |  |  |
| **Rurality^a^, %** |  |  |  |  |  |  |
| **Major urban** | 65.8 | 64.2 | 0.04 | 65.8 | 66.3 | 0.01 |
| **Non-major urban** | 26.7 | 26.4 | 0.01 | 26.7 | 26.8 | 0.00 |
| **Rural** | 7.5 | 9.4 | 0.07 | 7.5 | 7.0 | 0.02 |
| **Very high users of the healthcare system^b^, %** | 64.1 | 39.4 | 0.51 | 64.0 | 65.3 | 0.03 |
