# Supplementary material for: A population-based matched cohort study examining the mortality and costs of patients with community-onset Clostridium difficile infection identified using emergency department visits and hospital admissions
Source: PLoS One. 2017 Mar 3;12(3):e0172410. doi: 10.1371/journal.pone.0172410 (PMC5336215; doi:10.1371/journal.pone.0172410)
Supplement: S5 Table — Confidence intervals not reported, however, none of the confidence intervals crossed zero. CDI—C.difficile infection. NA—not applicable. (DOCX) [file pone.0172410.s005.docx]

|  | **n matched pairs** | **Costs unadjusted for survival** | | | **Costs adjusted for survival** | | |
| --- | --- | --- | --- | --- | --- | --- | --- |
|  |  | **Cumulative**  **30-day costs** | **Cumulative**  **180-day costs** | **Cumulative**  **1-year costs** | **Cumulative**  **1-year**  **costs, undiscounted** | **Cumulative**  **2-year**  **costs, undiscounted** | **Cumulative**  **3-year**  **costs, undiscounted** |
| **Overall** | 6,437 | $7,434 | $12,517 | $13,217 | $10,700 | $13,312 | $15,812 |
| **Sex** |  |  |  |  |  |  |  |
| **Female** | 4,192 | $7,437 | $12,177 | $12,682 | $10,746 | $13,468 | $15,984 |
| **Male** | 2,245 | $7,428 | $13,151 | $14,217 | $10,594 | $12,995 | $15,457 |
| **Age group** |  |  |  |  |  |  |  |
| **Children (≤18 years)** | 237 | $4,094 | $7,447 | $9,893 | $8,146 | $9,887 | $11,627 |
| **Adults (19-64 years)** | 2,466 | $5,162 | $9,071 | $10,918 | $9,194 | $11,517 | $14,005 |
| **Older adults (≥65 years)** | 3,734 | $9,147 | $15,114 | $14,947 | $11,099 | $13,548 | $15,687 |
| **Year of CDI diagnosis** |  |  |  |  |  |  |  |
| **2003** | 469 | $6,038 | $10,496 | $11,796 | NA | NA | NA |
| **2004** | 673 | $6,205 | $9,792 | $9,798 | NA | NA | NA |
| **2005** | 847 | $5,640 | $8,755 | $9,225 | NA | NA | NA |
| **2006** | 766 | $6,615 | $10,651 | $10,450 | NA | NA | NA |
| **2007** | 893 | $7,762 | $12,553 | $13,301 | NA | NA | NA |
| **2008** | 965 | $8,774 | $16,027 | $16,929 | NA | NA | NA |
| **2009** | 908 | $8,397 | $13,918 | $15,139 | NA | NA | NA |
| **2010** | 916 | $8,709 | $15,469 | $16,566 | NA | NA | NA |
| **Survivorship** |  |  |  |  |  |  |  |
| **Short-term** | 1,366 | $12,324 | $19,864 | $15,085 | $15,333 | NA | NA |
| **Long-term** | 5,071 | $6,117 | $10,538 | $12,714 | $13,105 | $16,484 | $19,708 |
